# The Research Centers in Minority Institutions (RCMI) Consortium: A Blueprint for Inclusive Excellence

**DOI:** 10.3390/ijerph18136848

**Published:** 2021-06-25

**Authors:** Elizabeth O. Ofili, Daniel Sarpong, Richard Yanagihara, Paul B. Tchounwou, Emma Fernández-Repollet, Mohamad Malouhi, Muhammed Y. Idris, Kimberly Lawson, Nadine H. Spring, Brian M. Rivers

**Affiliations:** 1 Community Health and Preventive Medicine and the RCMI Coordinating Center, Departments of Medicine, Morehouse School of Medicine, Atlanta, GA 30310, USA; myidris@msm.edu (M.Y.I.); klawson@msm.edu (K.L.); nspring@msm.edu (N.H.S.); brivers@msm.edu (B.M.R.); 2 Center for Minority Health and Health Disparities for Research and Education, College of Pharmacy, Xavier University of Louisiana, New Orleans, LA 70125, USA; dsarpong@xula.edu; 3 Department of Pediatrics, John A. Burns School of Medicine, University of Hawaii at Manoa, Honolulu, HI 96813, USA; ryanagih@hawaii.edu; 4 RCMI Center for Health Disparities Research, Jackson State University, Jackson, MS 39217, USA; paul.b.tchounwou@jsums.edu (P.B.T.); mohamad.malouhi@rtrn.net (M.M.); 5 Center for Collaborative Research in Health Disparities, Medical Sciences Campus, University of Puerto Rico, San Juan, PR 00936, USA; e.fernandez@upr.edu

**Keywords:** RCMI, inclusive excellence, workforce diversity, health equity, realist evaluation

## Abstract

The Research Centers in Minority Institutions, (RCMI) Program was established by Congress to address the health research and training needs of minority populations, by preparing future generations of scientists at these institutions, with a track record of producing minority scholars in medicine, science, and technology. The RCMI Consortium consists of the RCMI Specialized Centers and a Coordinating Center (CC). The RCMI-CC leverages the scientific expertise, technologies, and innovations of RCMI Centers to accelerate the delivery of solutions to address health disparities in communities that are most impacted. There is increasing recognition that the gap in representation of racial/ethnic groups and women is perpetuated by institutional cultures lacking inclusion and equity. The objective of this work is to provide a framework for inclusive excellence by developing a systematic evaluation process with common data elements that can track the inter-linked goals of workforce diversity and health equity. At its core, the RCMI Program embodies the trinity of diversity, equity, and inclusion. We propose a realist evaluation framework and a logic model that integrates the institutional context to develop common data metrics for inclusive excellence. The RCMI-CC will collaborate with NIH-funded institutions and research consortia to disseminate and scale this model.

## 1. Introduction

The National Academies report, titled “Rising Above the Gathering Storm: Energizing and Employing America for a Brighter Economic Future”, noted that, in order for the United States (U.S.) to maintain global leadership and competitiveness in science and technology, it must: (1) invest in research; (2) encourage innovation; and (3) grow a strong, talented, and innovative science and technology workforce [1]. Research suggests that the cultural diversity of a nation’s workforce is a key factor in its ability to innovate and compete in a global economy [2,3,4,5]. Although the STEMM (science, technology, engineering, mathematics, and medicine) workforce of the U.S. has grown more diverse over time, its numbers are still far below the level of diversity represented in the general population [6]. Tracking the impact of this underrepresentation is critical, given the imminent transition toward a non-White majority in the U.S. It is increasingly recognized that the educational outcomes and STEMM readiness of students of color will have direct implications on the nation’s economic growth, national security, and global prosperity [7]. Fortunately, the nation has a major asset in achieving this goal: the more than 700 minority-serving institutions (MSIs) that enroll nearly 30% of all undergraduates in the U.S. higher education system, the vast majority of whom are students of color [8,9].

This paper provides evidence of the impact of the National Institutes of Health (NIH) investments in the Research Centers in Minority Institutions (RCMI) Program, and, the premise that the RCMI Coordinating Center (RCMI-CC) is well positioned to evaluate, disseminate and scale NIH’s Diversity, Equity and Inclusion (DEI) initiatives.

### 1.1. The Challenge of Achieving Diversity in the Biomedical Research Workforce

The NIH has long recognized that achieving diversity in the biomedical and behavioral research workforce is critical to ensuring that the best and brightest minds have the opportunity to contribute to the achievement of our national research goals. The RCMI Program was established in 1985 in response to committee report language (House Report 98-911) attached to H.R. 6028, the Departments of Labor, Health and Human Services, and Education and Related Agencies Appropriation Act, 1985, to “establish research centers in those predominantly minority institutions which offer doctoral degrees in the health professions or the sciences related to health” [10,11]. Subsequent legislation (H.R. 3010) upheld and further recognized the critical role of the RCMI Program and encouraged the NIH to strengthen participation from minority institutions and resources available for this area.

The Department of Health and Human Services (DHHS) action plan to reduce racial and ethnic health disparities [12] and the National Institute on Minority Health and Health Disparities (NIMHD) 2021–2025 Strategic Plan emphasize the importance of a diverse workforce [13]. A growing field of investigation has unveiled the potential of a diverse workforce to improve health care access, increase patient satisfaction, and ensure culturally competent care by adequately addressing social determinants that impact health during medical interactions with patients [14].

Despite long-standing efforts by the NIH and other entities across the biomedical and behavioral research landscape to increase the number of scientists from underrepresented groups [15,16], Ginther and colleagues reported a disturbing discrepancy in success rates for research grant (such as R01) applications between White and Black applicants, even after controlling for numerous observable variables. Following the recommendation from the Advisory Committee to the NIH Director, the NIH established the Diversity Program Consortium (DPC) awards, which includes Building Infrastructure Leading to Diversity (BUILD) and National Research Mentoring Network (NRMN) at the National Institute of General Medical Sciences (NIGMS), coordinated by the newly established Chief Officer for Scientific Workforce Diversity (COSWD) [15,17,18,19]. Recent DPC analysis shows that Black applicants had modest improvement in applications and funding rate for R01-equivalent awards, but the numbers remain very low. In 2013 and 2020, Blacks submitted 425 and 703 applications vs. 16,918 and 19,919 for Whites. The funding rate increased from 12.2% to 23.6% for Blacks vs. 21.8 to 31.3% for Whites [17,20,21]. The RCMI Consortium contributed to this work through the NRMN, however such modest progress underscores the structural and systemic nature of the challenge, and the urgent need to scale and sustain evidence-based approaches of successful interventions in the career advancement of Black scientists.

The COVID-19 pandemic added to the urgency, by uncovering glaring health inequities and the devastation of structural racism on the health of African Americans, Latinx, Native Americans, Native Hawaiians, and Pacific Islanders [22].

### 1.2. The Impact of the RCMI Consortium on Workforce Diversity and Health Equity

At its core, the RCMI Consortium embodies the trinity of diversity, equity, and inclusion (DEI). The central role of the RCMI Consortium in addressing the NIH-linked objectives of workforce diversity and health equity was highlighted in three keynote presentations delivered at the RCMI Translational Science 2017 Conference in Washington, DC. During the opening plenary keynote, NIH Director Dr. Francis S. Collins, recognized the RCMI community as the brain trust to help develop novel solutions on health disparities [10]. During his plenary address, Dr. Eliseo Pérez-Stable, NIMHD Director, described the importance of addressing the levels of influence of health disparities across domains from the biological to the sociocultural environment including the health care system [10]. At the closing plenary, Dr. Lawrence A. Tabak, NIH Principal Deputy Director, noted the central role of the RCMI Program in the strategic plan of NIH [10].

An analysis of 18 RCMI Centers with active awards between 2002 and 2015 documented RCMI impact, as published in our seminal paper, titled: The Research Centers in Minority Institutions (RCMI) Translational Research Network: Building and Sustaining Capacity for Multi-Site Basic Biomedical, Clinical and Behavioral Research [10]. This publication described the impact of the RCMI Consortium on collaboration and scientific discovery, based on research funding, publications, patents, and return on investment: the RCMI Consortium converted approximately $900 million in overall program funding to almost $4 billion in additional research awards. The RCMI impact on workforce diversity was demonstrated: Over 22,000 science and health professional degrees were awarded; based on 2002 and 2012 data from the National Science Foundation, this represents one in four degrees awarded to African Americans and Hispanics during those years [10,23].

Similarly, RCMI Clinical Research Centers (CRCs) at Charles R. Drew University, Howard University, Meharry Medical College, Morehouse School of Medicine, University of Hawaii at Manoa, and University of Puerto Rico Medical Sciences Campus collectively enrolled over 16,000 diverse research participants between 2008 and 2016. There was broad representation across health disparity populations, including three quarters women; almost half were Black or African Americans; one third were Hispanics. Native Hawaiians, Whites, Asians, and Native American/Alaska Native were also represented in these clinical research studies. These clinical research centers also supported the training and career development of over 600 new clinical and translational research scholars over the same time period [10,24,25]. These clinical research centers demonstrated the importance of Community Advisory Boards, community and health system partnerships, in meeting the RCMI Consortium goals of recruiting and retaining diverse research participants. The RCMI Consortium demonstrated impact on the diversity of the nation’s biomedical research workforce while advancing scientific discovery and improving the health of minority communities across the health equity spectrum.

This manuscript will describe how the RCMI Consortium will build on its track record by developing and implementing consortium wide data collection processes and evaluation framework that will serve as a best practice for NIH DEI initiatives.

The overarching purpose of this work was to understand how a context-based evaluation framework can serve as a blueprint for establishing data standards and common metrics for inclusive excellence (IE). We explored how the centralized data model of the RCMI Consortium can support the evaluation of the RCMI U54 Centers, and ultimately to disseminate and scale the RCMI DEI model.

We hypothesized that the RCMI Consortium of institutions and the communities they serve provide a context-based and health equity-centered lens for a realist evaluation (RE) framework of IE.

Rationale: Despite the urgent need for diversity in NIH-funded institutions, data confirm that institutional cultures lacking the necessary elements of inclusion and equity consistently send the message that certain groups do not belong in science [26,27,28,29]. IE requires scientific environments that cultivate and benefit from a full range of talents, across the breadth of NIH-funded institutions, thus ensuring that the most talented researchers are recruited, supported, and advanced to become competitive investigators [30]. IE requires an understanding of what works for whom, and in what context. We propose that evaluating IE should integrate institutional context, individual career outcomes, as well as system level outcomes for DEI. Our premise recognizes that the dual meaning of IE should be incorporated in its evaluation framework: (i) excellence in the process of inclusion, which examines how well an institution is achieving its specific objectives of diversity; (ii) measuring the impact of a collaborative vibrant and diverse workforce on research excellence. This premise is consistent with the National Academy of Science Engineering and Medicine (NASEM) report’s recommendation that DEI interventions seeking to sustain institution and system-level change must address the interconnected components of a complex system of rewards and incentives by: (1) driving transparency and accountability; (2) adopting data-driven approaches to address underrepresentation; (3) rewarding, recognizing and resourcing diversity, equity, and inclusion; (4) filling knowledge gaps [31].

### 1.3. Theoretical Framework

**Figure 1:**
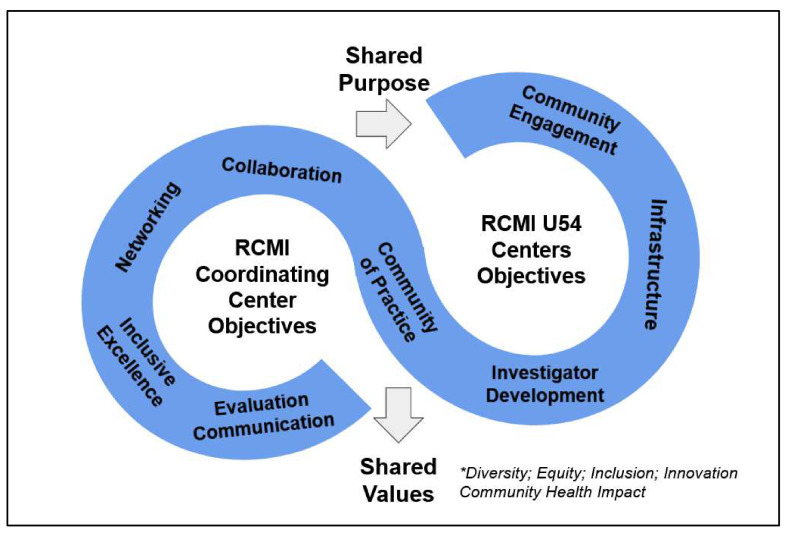
The inter-linked objectives of the RCMI Coordinating Center and the RCMI U54 Centers is anchored in a shared purpose, and centered on shared values of diversity, equity, inclusion, innovation and community health impact.

The theoretical framework of the RCMI Consortium is the Community of Practice and Stakeholder Engagement model [32,33], which is anchored in the shared purpose of the RCMI Consortium (Figure 1). The RCMI Consortium consists of the independently funded RCMI-CC, which is tasked with working with key personnel at each of the competitively funded RCMI Specialized Centers (U54 Centers) and NIMHD staff to help the U54 Centers collectively achieve their objectives to: (1) enhance institutional research capacity within the areas of basic biomedical, behavioral, and/or clinical research; (2) enable all levels of investigators to become more successful in obtaining competitive extramural support, especially from NIH, particularly on diseases that disproportionately impact minority and other health disparity populations; (3) foster environments conducive to career enhancement with a special emphasis on the development of early career investigators; (4) enhance the quality of all scientific inquiry and promote research on minority health and health disparities; and (5) establish sustainable relationships with community-based organizations that partner with RCMI U54 Centers. The Community of Practice and Stakeholder engagement framework enables ongoing needs assessment and the sharing of best practices for the continuous learning and improvement of each RCMI U54 Center.

The RCMI-CC goals include evaluating the RCMI U54 Centers and the RCMI Consortium. The RCMI-CC integrates the Community of Practice and Stakeholder engagement in its needs assessment as described in the methods section. Given the diversity of research resources across the RCMI Consortium institutions, we propose the realist evaluation (RE) framework [34,35,36], which focuses on identifying program context, mechanisms, and outcomes to determine what works for whom under what circumstances. As such, RE does not simply assess program outcomes, but explores configurations of pre-program resources and cultures (contexts) [34,35,36]. The RCMI-CC RE logic model centers on shared values of diversity, equity, inclusion, innovation, and community health impact (Figure 1). The collaboration culture of the RCMI Consortium acknowledges that no single individual or institution has all the knowledge, skills, and resources needed to address DEI and the pressing health issues facing our nation [37,38,39,40,41,42,43,44].

**Figure 2:**
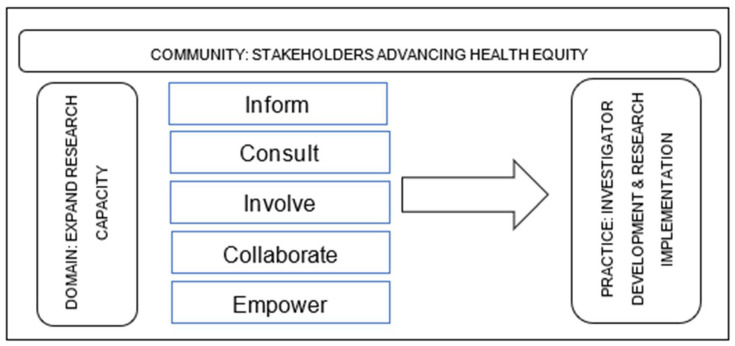
The RCMI Community of Practice and Stakeholder-Engagement Model.

The Community of Practice Stakeholder-Engagement Framework [45,46] is a systematic logical, practical, and measurable approach to defining and aligning RCMI-CC objectives with RCMI U54 Centers objectives on investigator development and research implementation (Figure 2). RCMI stakeholders are U54 Centers principal investigators, core directors, research scientists/mentors, early-stage investigators, clinicians, community and health system partners, and NIMHD project scientists and program officials.

### 1.4. How the Study Is Unique and Builds on Existing Literature

Current NIH diversity programs lack the evidence for a standardized DEI evaluation framework [31]. This presents a challenge for the dissemination and scaling successful DEI models. Our work addresses the following inter-connected four pillars, recommended by the NASEM for DEI interventions that could sustain IE at the institution and system-level: (1) transparency and accountability: institutions must articulate and deliver on measurable goals and benchmarks that are regularly monitored and publicly reported; (2) data-driven approaches to address underrepresentation: by segmenting barriers by discipline and career stage, and using disaggregated data collection, analysis, and monitoring as the basis for constructing specific interventions within the unique context of each institution; (3) rewarding, recognizing and resourcing DEI efforts by institutions are often hindered by a lack of sufficient resources and by the expectation that individuals, particularly people of color and women, who are most affected by these issues, will assume a leadership role in promoting positive change without appropriate compensation, authority, or promise of reward or recognition; (4) filling knowledge gaps to support scholarly work on DEI. Our goal is to develop a replicable RE logic model using standardized data elements and metrics that can be adapted across NIH funded institutions seeking to implement and transparently report on inclusive excellence.

With funding support, the RCMI-CC will collaborate with NIH-funded research networks, such as the Clinical and Translational Science Award (CTSA) and Institutional Development Award (IDeA) consortia, to disseminate and scale the adoption of the RCMI DEI model across various institutional contexts, from research-intensive to resource-limited settings [37,38,39,40]. The evaluation model provides a framework to define, standardize, and adapt IE to meet the needs of diverse investigators and institutions. We anticipate that our proposed logic model will need to be continuously refined as more institutions adapt the framework.

We have implemented a centralized RCMI database for baseline and longitudinal RCMI investigators data collection. We are also integrating the open-source central data repository, Dataverse [47], to share and archive common data elements across RCMI consortium members, as well as research data from RCMI-related collaborations. Our long-term goal is to utilize AI algorithms to define context specific investigator outcomes of DEI interventions, which could be applicable across NIH funded institution settings.

## 2. Methods

### 2.1. RCMI U54 Centers Needs Assessment

The needs assessment was conducted between 18 September and 11 December 2019, and designed to define the priorities, and identify resource gaps, for each RCMI U54 Center. Each U54 Center identified areas of expertise, and opportunities to strengthen its Center’s capacity for inter-institutional research collaboration, by prioritizing access to the following resources: research studio/investigator development; biostatistics expertise; bioinformatics expertise; community engagement expertise; human subjects recruitment expertise; letters of support; finding collaborators (for multi PI proposals, and co-authors on publications); technology transfer; institutional review board; samples/biorepository/laboratory methods; industry clinical trials; NIH multi-site studies; collaborative pilot project funding; RCMI Program National Conference; minority health and health disparities research training; national research mentoring network resource.

### 2.2. Community of Practice and Stakeholder Engagement

**Figure 3:**
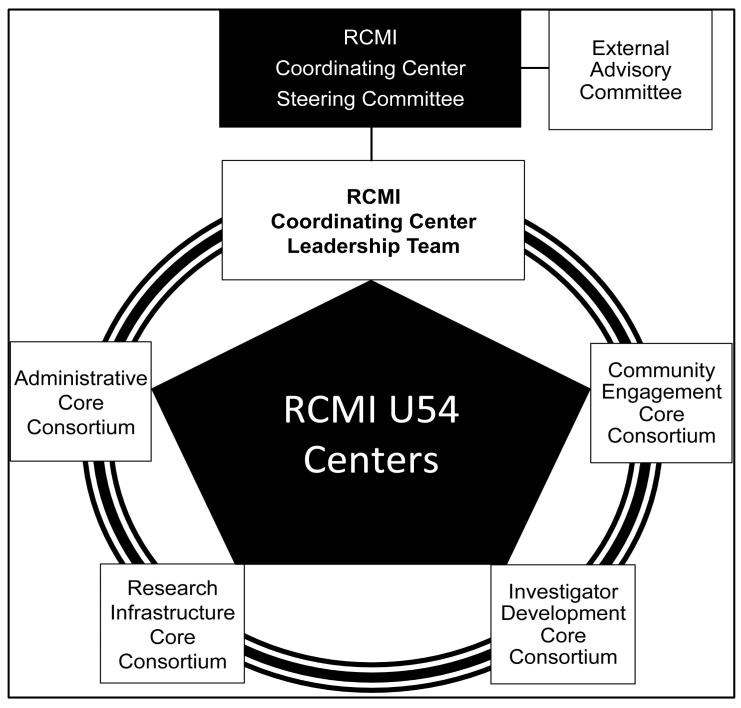
The Organization of the RCMI Consortium.

The organization of the RCMI Consortium, its stakeholders, and governance are shown in Figure 3, and described here. The Community of Practice and stakeholder engagement is enabled by the organization of the RCMI Consortium, which includes a representative governing Steering Committee. The Steering Committee approves major collaborations and ensures compliance with NIH policies on multi-site collaboration, data sharing, regulatory and publications. Each Core Consortium represents a key function of the RCMI U54 Center, and includes the respective core directors from across the U54 Centers, and the RCMI-CC multi-PI (MPI) with responsibility for coordinating the activity of that core/key function.

For example, the Administrative Core Consortium includes the RCMI-CC Contact PI Steering Committee Chair and all the U54 Centers principal investigators/program directors. The Evaluation Consortium resides within the Administrative Core of each RCMI U54 Center and the RCMI-CC. The Community Engagement Core Consortium, Investigator Development Core Consortium, and Research Infrastructure Core Consortium are similarly configured to include the directors from the respective cores and the RCMI-CC MPIs.

The objective of the stakeholder engagement was to: (1) develop a theory-based evaluation protocol, agree on common metrics, and standardize data collection process; (2) engage RCMI Centers directors in a continuous process improvement and sharing of best practices on investigator development, community engagement, resource sharing, and collaborations, relevant to their respective U54 Center goals and scientific activities; (3) agree on a process to monitor and share outcomes annually. We conducted two full days of Consortium-wide research retreat and strategy sessions followed by breakout sessions. The strategy and breakout sessions were followed by full day workshops for the respective Consortium Core leaders. The workshops were conducted during the RCMI Annual Scientific Conference, with full participation of all 21 active U54 Centers. Each Core Consortium provided direct input to the RE logic model being developed by the Tracking and Evaluation Consortium.

We used a modified Delphi method [48], where the RCMI Core Consortium acted as expert panels to: (1) remove duplicate items; (2) sort into categories aligned with RCMI Center program goals and DEI shared values; (3) retain items that were most conceptually clear and aligned with DEI literature. Disagreements were resolved through discussion until consensus was reached. During the strategy breakout sessions and full day workshops, each RCMI Core Consortium identified national programs that are relevant to their respective goals, and where appropriate, adopted metrics from the national program. For example, the Investigator Development Core Consortium adopted grant success and research development metrics from the NRMN [20,21]. Similarly, the Community Engagement Core Consortium adopted metrics from the NIH All of Us Program [49].

Given that evaluation, common metrics, and data standardization are goals of this work, we referenced evaluation programs, as well as lessons learned from the CTSA program [50,51,52,53], to develop a logic model based on the RE theoretical framework [34,35,36]. The logic model integrates a DEI lens, in order to connect research development activities to investigator outcomes and community health impact [49,50].

### 2.3. RCMI Consortium Centralized Database for Collaboration and Data Collection

To maximize the potential for improved inter-institutional engagement and collaboration, the RCMI-CC established a centralized database to support research collaboration and data collection across the RCMI Consortium. The searchable database can be used for finding investigators, and groups with specific areas of expertise, as well as search publications [10,38,39]. The RCMI Consortium established a centralized database that uses Pubmed and NIH RePORTER to accomplish the data collection objectives and publication outcomes, including inter-institutional collaborations, for all investigators as well as research expertise across the consortium to: (1) track productivity (specifically, publications and grants, with visualization tools to track inter-institutional collaborations; (2) streamline the process for finding new collaborators; (3) provide a platform to standardize data collection across the consortium; (4) leverage data-mining techniques to identify inter-institutional passive networks and shared research connections across the consortium; (5) lower the cost of operation by eliminating duplicate efforts and aligning resources for data collection.

Capitalizing on a centralized research-centric system to track productivity is essential to streamline the process for tracking research productivity and inter-institutional collaborations, especially when working with large research networks such as the RCMI Consortium. Using the centralized database as a systematic approach to track productivity at various levels yields consistent, objective, and standardized results across all the RCMI U54 Centers. Two of the most used key performance indicators (KPI) to assess scientific productivity are the number of publications and the number of awarded grants, both of which are routinely (quarterly) captured by the RCMI-CC centralized database. By harnessing the power and capabilities of the underlying relational database management system (RDBMS) of the centralized database, the RCMI-CC will be able to produce a quantitative representation of research activities (publications and grants) over time, at the individual (researcher) level as well as at the institution and consortium levels. In addition to the number of corresponding publications/awards, the centralized database captures other data elements such as: (1) How relevant the concepts of publications to the overall topic? (2) How long ago the publications were written? (3) Was the person the first or senior author, and how many other people have written about the same topic? The data can also identify shifts in research focus over time or reveal gaps in research productivity. The centralized database provides visualizations and timelines that show the dates of publications, top concepts, and concept clouds to emphasize keywords that make a person’s research unique, illustrating changes in the primary topics over time. Other visualizations display cluster and radial graphs of authors, co-authors, and co-authors of co-authors. The graphs illustrate the proportional number of a person’s publications and a proportional number of publications that they share with co-authors.

In addition to the centralized database, we are also implementing an open-source central data repository, Dataverse [47], to share and archive common data elements across RCMI consortium members as well as research data from RCMI-related collaborations. There are several features that make Dataverse unique: First, the platform has already implemented Findable Accessible Interoperable Reusable (FAIR) data principles into the platform [54]. This includes generating DOIs automatically for global persistent IDs, citation and domain-specific metadata, and an access and usage control system. Second, the platform also includes existing integrations for GUI-based tools for searching, data exploration, and quantitative analysis as well as future integrations that focus on data privacy. This includes DataTags that outline security features and access requirements for file handling and a private data sharing interface that leverages novel differential privacy methods to release privacy preserving statistics through a user interface that withholds information about individuals in the dataset [55]. Summary reports and analyses, including both evaluations of IE as well as research collaborations, will be uploaded to Dataverse to ensure reproducibility and promote data sharing [56,57].

## 3. Results

### 3.1. RCMI Consortium Institutions with Active U54 Center Awards

The 21 RCMI grantee institutions are located across 12 States, the District of Columbia, and Puerto Rico (Table 1). Each RCMI Center maintains trusted relationships and partnerships with the communities they serve. These communities are most severely impacted by health inequities and include racial/ethnic minorities, i.e., African Americans or Blacks; Hispanics or Latinx; Native Americans and Alaska Natives; Native Hawaiians and Pacific Islanders. RCMI grantee institutions also serve underserved rural populations and sexual/gender minorities. The students and investigators of 21 institutions, the 14 geographical locations of RCMI, and the communities served by RCMI undergird the RCMI DEI pillar of IE.

### 3.2. RCMI U54 Centers Prioritize Community Engagement Expertise and Investigator Development for Research Collaboration

The results of the needs assessment showed that all (100%) RCMI U54 Centers prioritized community engagement expertise. Other prioritized resources were: 94% letters of support; 89% pilot project funds; 83% access to biostatistics expertise; bioinformatics expertise and finding collaborators for multi-PI research funding opportunities and publications; 78% investigator development research studios, NRMN resources, RCMI Annual Conference, and health disparities training; 72% technology transfer and innovation; 67% human subjects resource/institutional review board. These results bolster the DEI focus of the RCMI Evaluation framework and will be prioritized for common data elements in the logic model. The priorities identified in the needs assessment are included as data elements in the research resources and knowledge resources, which are inputs for the RE logic model. The needs assessment also show that RCMI Consortium members are interested in collaborations with RCMI and non-RCMI investigators. The dissemination and implementation of the RCMI DEI evaluation framework will benefit from this robust collaboration capacity. The fostering of collaboration among young and early investigators, seasoned investigation internal and external to each U54 RCMI, and bi-directional relationship between academic and community partners underscore the RCMI Program’s commitment to inclusion and community focused priorities that impact health outcomes.

### 3.3. Registered Investigators in the Centralized RCMI Profiles Database

**Figure 4:**
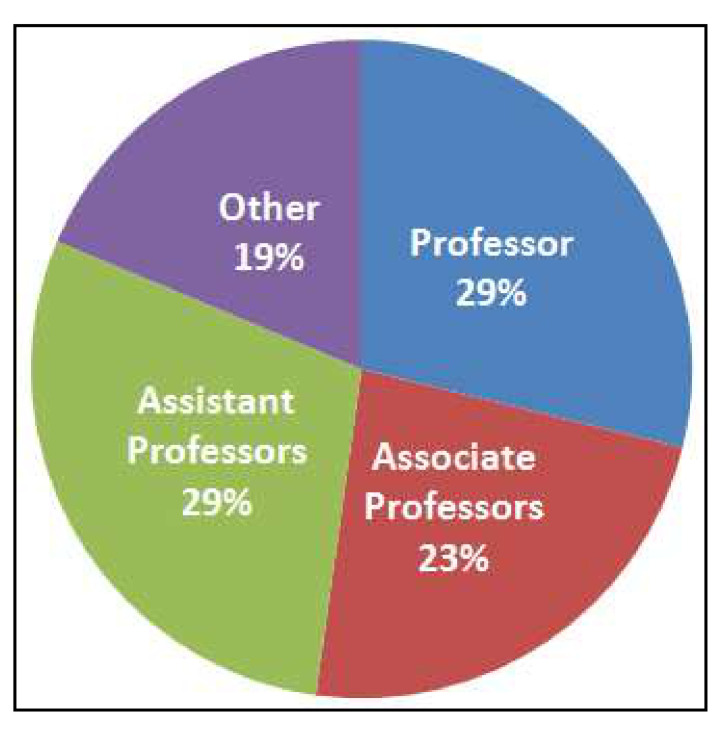
Distribution of RCMI Consortium investigators by academic rank.

The centralized database includes existing active, and newly registered investigators. The newly registered investigators were on-boarded between January through 22 April 2021. Table 2 shows the number of active investigators. These results suggest significant engagement of RCMI Consortium investigators. Inter-institutional unique publications reveal 560 investigators made 2284 direct connections and research collaborations. Distribution of investigators by academic rank are shown in Figure 4. Table 3 shows publications per institution. Tracking and evaluating common metrics across the U54 RCMI Centers is essential in demonstrating the scientific and workforce development successes of the RCMI Program consistent with IE.

### 3.4. Sample of Research Domains of Early Stage Investigators

**Figure 5:**
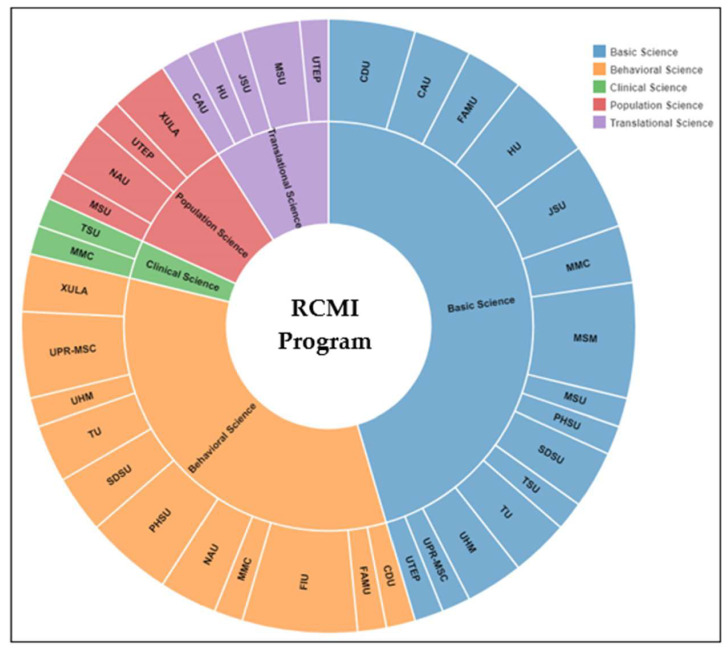
Scientific domains of abstracts presented by ESIs from RCMI U54 Centers. See Table 3 for abbreviated names of RCMI institutions.

This sample is from scientific abstracts accepted for presentation at the 2021 RCMI Annual Conference. Figure 5 shows the research domain of 100 abstracts presented by early-stage investigators (ESI). This snapshot demonstrates the different areas of scientific emphasis across the RCMI Consortium, with research domains of basic science; behavioral science; clinical science; populations science; translational science. Note that this is a limited sample of abstracts presented at the RCMI Virtual Conference, where investigator participation was limited due to COVID-19 and the Virtual environment. It does not represent the entirety of research across RCMI institutions. The full impact of the research conducted by RCMI Consortium investigators is documented in peer-reviewed publications (Table 3). The scientific contributions of the RCMI investigators, highlighted in Table 3 and in Figure 5, speak to the innovation, equity, and diversity in the research conducted by this community of investigators with varied levels of research infrastructure and capacity and research culture.

### 3.5. Use Case: RCMI Consortium Collaboration with the National Research Mentoring Network (NRMN)

Our group published the paper titled “Using a Virtual Community (the Health Equity Learning Collaboratory) to Support Early-Stage Investigators Pursuing Grant Funding” [20]. This work demonstrated the capacity of the RCMI Consortium to support the NIH DPC, by implementing the NRMN Research Resources and Outreach Core to recruit diverse early-stage investigators (ESIs) from RCMI and non-RCMI institutions. The unique training environment in the Health Equity Learning Collaboratory enabled RCMI and non RCMI investigators from diverse backgrounds to be successful with grant submissions, and 26% funding rate [20,21]. This mentoring approach continues to support ongoing NRMN and DPC interventions through a U01 awarded by the National Institute of General Medical Sciences [58]. The success of RCMI and non RCMI scholars using the NRMN Health Equity Collaboratory [20] is an example of a tailored intervention that is generating real-time qualitative data to support innovative data collection and analysis methods (see Future Plans).

### 3.6. RCMI-CC Realist Evaluation (RE) Logic Model

The RE logic model’s inputs, activities, outputs, outcomes, community and health equity impact will track the four pillars that drive sustainable interventions (see Table 4) [34,35,36]. The common data elements will ensure: (1) transparency and accountability; (2) adopting data-driven approaches to address underrepresentation; (3) rewarding, recognizing, and resourcing diversity, equity, and inclusion; (4) filling knowledge gaps. The RCMI Consortium will prioritize dissemination through publication in peer-reviewed journals.

Influence of the external environment including at the agency, state, region, or national policies may be standardized where feasible for purposes of comparative analysis.

## 4. Discussion

In this article, we present the design and RE logic model for a context-based evaluation framework of IE, by developing a systematic process with common data elements that align with the inter-linked goals of workforce diversity and health equity. We demonstrate that the mission, goals, and track record of the RCMI Program positions it to develop and standardize common data elements relevant to IE. The RCMI-CC centralized database and Dataverse open-source data-sharing model support collaboration capacity, and support users to disseminate and scale the model. The RE places our study in the larger context of NIH DEI initiatives. The logic model, common data elements, and data standardization will serve as a blueprint for adapting the model to diverse NIH-funded institutions, from research intensive to resource limited settings. The NRMN use case demonstrates the capacity of the RCMI Consortium to support national initiatives on DEI. The RCMI-CC governance and evaluation objectives support future collaboration that can disseminate and scale the RE model. By developing baseline common data elements that can apply across institutions, as well as investing in the Dataverse platform for secure and privacy protected data sharing, the RCMI-CC is establishing a RE grounded logic model for transparent and comparative evaluation of IE at RCMI Centers as well as across NIH funded institutions, regardless of the institution size, resources, public, or privately funded. The RE logic model and common data elements serve to establish a transparent and accountable process to collect and refine baseline data that will support DEI and IE processes and outcomes.

This work aligns with the commitment and statement of NIH Director Collins, who declared, on 1 March 2021, that NIH stands against structural racism in biomedical research: “to those individuals in the biomedical research enterprise who have endured disadvantages due to structural racism, I am truly sorry. NIH is committed to instituting new ways to support diversity, equity and inclusion, and identifying and dismantling any policies and practices at our own agency that may harm our workforce and science”. Adding action to words, he announced a new NIH initiative called UNITE, unveiled a website on Ending Structural Racism [59], and issued a Request for Information (RFI) that seeks input on practical and effective approaches to address racial and health inequities.

### Future Plans

The evaluation framework and common metrics represent the first step in the RCMI DEI blueprint for IE. A next step is to complete the implementation of the model in coordination with RCMI U54 Centers. The next step will include establishing benchmarks for publication and research excellence outcomes, that incorporates the RE logic model resource inputs. Our goal is to standardize outputs, based on the baseline data that will populate the RE logic model (anticipated completion in 2022). We will publish this work, to support dissemination and scaling in collaboration with NIH research consortia, including CTSA, NRMN/DPC, and IDeA programs. In addition, we are exploring innovative data collection and analysis methods by leveraging the EQ collaboratory [20,21], to engage RCMI investigators in real time. We will implement ecological momentary assessments in the form of periodic open-ended prompts to collect qualitative data on investigator experiences and IE over time. These responses as well as other unstructured communications will be exported from the EQ collaboratory, linked to investigator metadata, and will be analyzed using statistical and machine learning techniques to supplement traditional evaluation methods [60].

## 5. Conclusions

This work confirms our hypothesis that the RCMI Consortium context-based and health equity-centered evaluation of IE can serve as a blueprint for NIH DEI initiatives. As the NIH embarks on efforts to address structural and systemic racism, DEI initiatives should prioritize context-based evaluation and common data elements that transparently track and publicize individual, institution, as well as system level outcomes. The RCMI Consortium welcomes a bidirectional outreach and collaboration with NIH-funded institutions and research consortia, as we seek to collectively engage and support the best and the brightest underrepresented investigators in biomedical research.

## Figures and Tables

**Table 1:** Actively funded RCMI U54 centers.

Charles R. Drew University, Los Angeles, CA.	Ponce Health Sciences University, Ponce, PR.
Clark Atlanta University, Atlanta, GA	San Diego State University, San Diego, CA.
Florida Agricultural & Mechanical University, Tallahassee, FL.	Texas Southern University, Houston, TX.
Florida International University, Miami, FL.	Tuskegee University, Tuskegee, AL.
Howard University, Washington, DC.	University of California, Riverside, Riverside, CA.
Jackson State University, Jackson, MS.	University of Hawaii at Manoa, Honolulu, HI.
Meharry Medical College, Nashville, TN.	University of Houston, Houston, TX, USA
Morehouse School of Medicine, Atlanta, GA.	University of Puerto Rico, Medical Sciences Campus, San Juan, PR.
Morgan State University, Baltimore, MD.	University of Texas at El Paso, El Paso, TX.
North Carolina Central University, Durham, NC.	Xavier University of Louisiana, New Orleans, LA.
Northern Arizona University, Flagstaff, AZ.	

**Table 2:** Registered investigators and collaborative publications.

Registered Investigators and Collaborative Publications	Number of Records
Number of existing records	1281
Number of newly registered investigators (January–April 2021)	974
Number of updated records	1240
Number of affiliated with active RCMI institutions	1959
Number of inter-institutional unique publications (2000–2021) 560 Investigators and 2284 direct connections	871
Total Number of Active Investigators	2252

**Table 3:** Number of Publications per Institution from 2000–2021.

Institution Name	Publications	Institution Name	Publications
Charles R. Drew University (CDU)	3256	Ponce Health Sciences University (PHSU)	678
City College of New York (CCNY) *	1961	San Diego State University (SDSU)	1221
Clark Atlanta University (CAU)	726	Texas Southern University (TSU)	798
Florida A&M University (FAMU)	1010	Tuskegee University (TU)	333
Florida International University (FIU)	1463	Universidad Central Del Caribe (UCC) *	877
Howard University (HU)	1938	University of California Riverside (UCR)	316
Hunter College (HC) *	2278	University of Hawaii at Manoa (UHM)	9668
Jackson State University (JSU)	1726	University of Houston (UH)	1162
Meharry Medical College (MMC)	2263	University of Puerto Rico (UPR-MSC)	3862
Morehouse School of Medicine (MSM)	2577	University of Texas at El Paso (UTEP)	1893
Morgan State University (MSU)	599	University of Texas at San Antonio (UTSA) *	4477
North Carolina Central University (NCCU)	550	Xavier University of Louisiana (XULA)	685
Northern Arizona University (NAU)	1098	**Total**	47,415

* Not currently funded by NIMHD as RCMI U54 Center. Note that RCMI Centers are competitively funded based on NIMHD award cycles. Investigators from institutions that have enrolled in the RCMI database are retained in the database. Each investigator’s institutional affiliation may be re-activated when that institution successfully competes for RCMI funding. However, only actively funded institutions are longitudinally tracked for RE logic model.

**Table 4:** The RCMI-CC Realist Evaluation (RE) Logic Model Integrates Institution Context.

Resources/Inputs	Scientific Activities	Scientific/DEI Outputs (Institution)	Outcomes	Community & Health Equity Impact
**Financial Resources** Financial Administration Pilot project funds	**Funding** Research Project Applications (new and continuing) Research Awards	**Scientific Outputs** New and renewing R01 New and renewing R01 equivalent New and renewing program awards Peer reviewed Publications Patent Disclosures	**Individual Investigator** Appointments and promotion Tenure track/equivalent Research awards Leadership Awards Mentoring awards Teaching Awards	**Health Disparity** **Population Impact** Practice Guidelines that impact health equity
**Infrastructure Resources** Research Space Research Infrastructure Mentoring/NRMN Training Career Development	**Collaborations** Multi-PI R01 Awards Multi-site Research Team based Publications			Community, Health Equity and Public Health Benefits Health promotion Population Health Public health impact
**Human Resources** Diverse faculty & Students URG/Women/LGBTQ Community partners Health system partners	**Community Engagement** Community partnered & Community led research Awards NIH & Industry Trials	**DEI Outputs** Doctoral degrees awarded to URG/Women/LGBTQ Training grants awarded to URG/Women/LGBTQ Health equity research awards Recruitment of URG participants	**RCMI Center &** **Institution** New Research Awards from NIMHD /NIH ICs Research Endowment	**Economic Benefits** Commercial products Financial savings (ROI)
**Knowledge Resources** Methodology Biological/Clinical materials and tools Information Services Health Technology	**Innovation & Regulatory** Research Ethics Research Compliance Incubator/SBIR Data Collection Data Analysis		**RCMI Consortium & National** Service on RCMI Steering Committee National Advisory Boards National Awards/Honors	**Policy and Legislation with Health Equity Lens** Advisory activities Policies and legislation
External Environment Influence: Structural and Systemic Racism; Politics; Health Equity; HHS/NIH Policy				

## Data Availability

The study did not report archivable data.
